# Correction: Group Size Effect on Cooperation in One-Shot Social Dilemmas II: Curvilinear Effect

**DOI:** 10.1371/journal.pone.0138744

**Published:** 2015-09-16

**Authors:** The PLOS ONE Staff

The URL in the Data Availability statement for this paper is incorrect. The correct URL is http://dx.doi.org/10.5061/dryad.j66q1. The publisher apologizes for the error.
